# Shared and divergent phase separation and aggregation properties of brain-expressed ubiquilins

**DOI:** 10.1038/s41598-020-78775-4

**Published:** 2021-01-11

**Authors:** Julia E. Gerson, Hunter Linton, Jiazheng Xing, Alexandra B. Sutter, Fayth S. Kakos, Jaimie Ryou, Nyjerus Liggans, Lisa M. Sharkey, Nathaniel Safren, Henry L. Paulson, Magdalena I. Ivanova

**Affiliations:** 1 Department of Neurology, University of Michigan, Ann Arbor, MI 48109-2200 USA; 2 Department of Neurology, Biophysics Program, University of Michigan, Ann Arbor, MI 48109-2200 USA; 3 Present Address: Neuroscience Graduate Program, Northwestern University Feinberg School of Medicine, Chicago, IL 60611 USA; 4 Present Address: Department of Neurology, Northwestern University Feinberg School of Medicine, Chicago, IL 60611 USA

**Keywords:** Biochemistry, Neuroscience

## Abstract

The brain-expressed ubiquilins, UBQLNs 1, 2 and 4, are highly homologous proteins that participate in multiple aspects of protein homeostasis and are implicated in neurodegenerative diseases. Studies have established that UBQLN2 forms liquid-like condensates and accumulates in pathogenic aggregates, much like other proteins linked to neurodegenerative diseases. However, the relative condensate and aggregate formation of the three brain-expressed ubiquilins is unknown. Here we report that the three ubiquilins differ in aggregation propensity, revealed by *in-vitro* experiments, cellular models, and analysis of human brain tissue. UBQLN4 displays heightened aggregation propensity over the other ubiquilins and, like amyloids, UBQLN4 forms ThioflavinT-positive fibrils in vitro. Measuring fluorescence recovery after photobleaching (FRAP) of puncta in cells, we report that all three ubiquilins undergo liquid–liquid phase transition. UBQLN2 and 4 exhibit slower recovery than UBQLN1, suggesting the condensates formed by these brain-expressed ubiquilins have different compositions and undergo distinct internal rearrangements. We conclude that while all brain-expressed ubiquilins exhibit self-association behavior manifesting as condensates, they follow distinct courses of phase-separation and aggregation. We suggest that this variability among ubiquilins along the continuum from liquid-like to solid informs both the normal ubiquitin-linked functions of ubiquilins and their accumulation and potential contribution to toxicity in neurodegenerative diseases.

## Introduction

The ubiquilins are highly homologous proteins that regulate multiple protein homeostasis pathways. Of the mammalian ubiquilins, UBQLN1, UBQLN2 and UBQLN4 are expressed in the brain and are associated to varying degrees with neurodegenerative diseases characterized by protein misfolding, aggregation and mislocalization. Mutations in UBQLN2 cause a rare familial X-linked neurodegenerative disease belonging to the amyotrophic lateral sclerosis/frontotemporal dementia (ALS/FTD) spectrum^1,2^. Both UBQLN1 and UBQLN2 colocalize with disease aggregates in ALS/FTD, Huntington’s disease, and to a lesser extent, the synucleinopathies^3,4^. A genetic variant of UBQLN4 has also been implicated in ALS, suggesting that UBQLN4, like UBQLN1 and UBQLN2, contributes to neurodegeneration^5^.

The involvement of the ubiquilin family in protein accumulation associated with neurodegenerative disease likely reflects their functions in protein quality control. All three brain-expressed ubiquilins contain a C-terminal ubiquitin-associated (UBA) domain, an N-terminal ubiquitin-like (UBL) domain and four stress-induced protein-like (STI) domains that mediate binding to molecular chaperones. The most well-characterized function for ubiquilins is as ubiquitin–proteasome shuttle factors^6,7^, but they have been implicated in other protein homeostasis pathways including autophagy and endoplasmic reticulum-associated protein degradation (ERAD)^8–12^.

Like many other proteins associated with neurodegenerative diseases^13–18^, UBQLN2 spontaneously phase separates to form condensates, or liquid droplets, in which proteins are concentrated yet remain mobile^13,19–21^. Liquid-like condensates are tightly regulated by cells and can provide sites for specific cellular functions^22–25^. Evidence for this includes a recent finding that UBQLN2 regulates the fluidity of protein–RNA complexes in FUS-related ALS/FTD^26^. At the same time, dysregulated condensate formation by various proteins leads to abnormal protein accumulation and disease^27,28^. Indeed, it has been reported that the cytosolic accumulation of UBQLN2 drives aggregation of TDP43 in the cytoplasm^21^.

In vitro, high salt concentrations promote UBQLN2 phase separation^19,20^. Large punctate UBQLN2 assemblies resembling liquid condensates form in cells overexpressing UBQLN2^13,21^. In particular, mutations in a proline-rich domain present in UBQLN2 mediate the propensity of UBQLN2 to form less mobile liquid condensates and fibrillar aggregates^13,20^, suggesting that dynamic changes to ubiquilin condensation may underlie toxicity in mutation-driven disease.

The liquid–liquid phase transition behavior and aggregation propensity of the other ubiquilins remain unknown. Because UBQLN1 and UBQLN2 form or co-localize with pathological deposits in disease^3,29^, and all three brain-expressed ubiquilins are highly homologous^30^, we sought to define their relative phase separation and aggregation properties. Through in vitro studies, cellular models, and analyses of human tissue, we show that the three brain-expressed ubiquilins differ in condensate formation and aggregation. The results support the view that these highly homologous proteins exhibit complex association behavior and suggest that an imbalance in phase transitions may play a role in neurotoxicity related to ubiquilin protein dysregulation.

## Results

### Among brain-expressed ubiquilins, UBQLN4 is particularly prone to self-assemble into fibrillar aggregates in vitro

**Figure 1:**
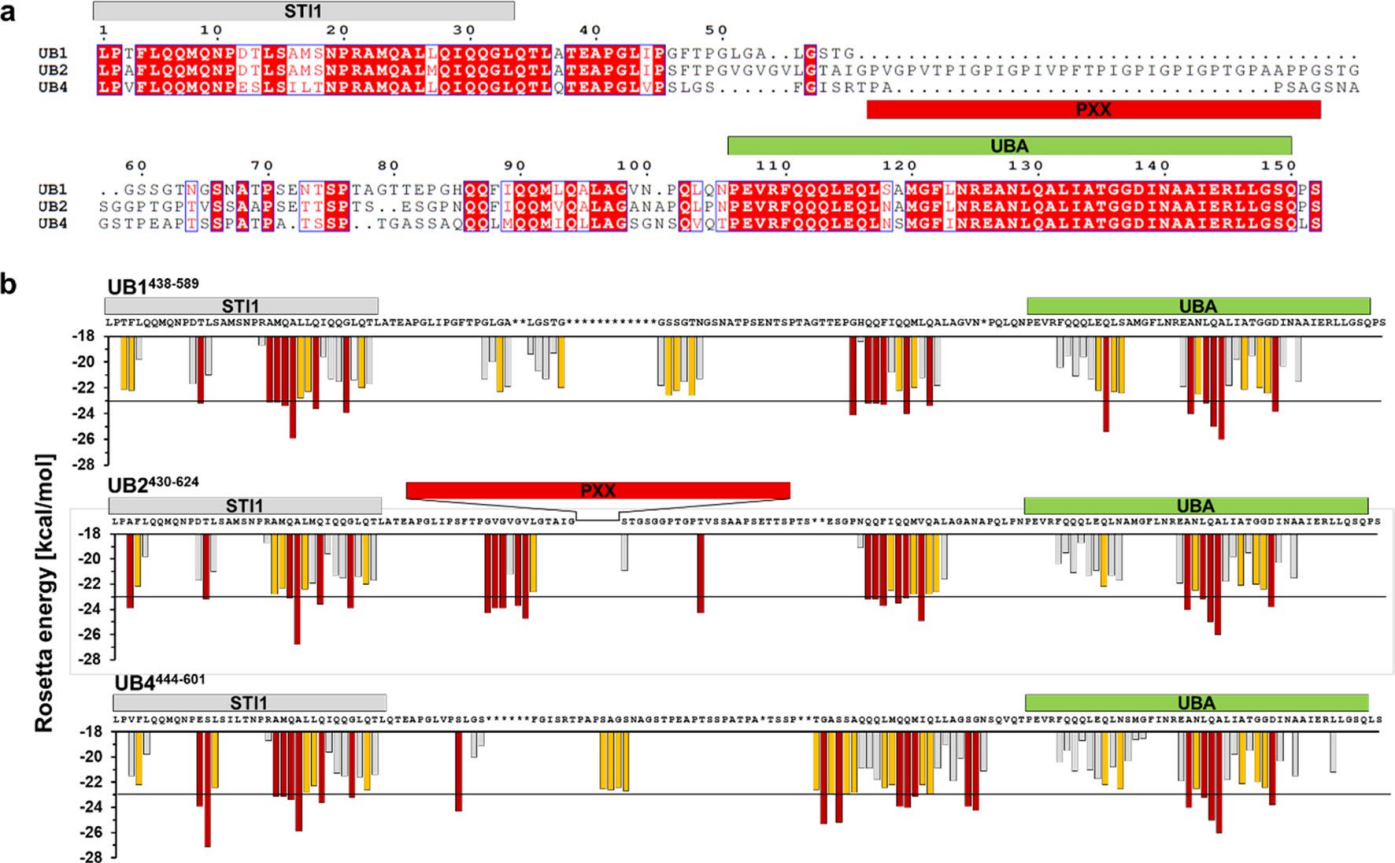
Sequence alignment and prediction of aggregation-prone regions of C-terminal ubiquilin constructs (UBQLN1^438–589^, UBQLN2^430–624^, and UBQLN4^444–601^) used for in vitro studies. (**a**) The UBA domains are highly conserved (93% identical) with the major sequence differences residing in the region between STI1-4 and UBA^30,51^. Identical residues are colored in red. (**b**) Zipper DB was used to predict regions that may drive aggregation of ubiquilin constructs^31^. In the histogram, each bar represents a six-residue-long segment. Segments with Rosetta energies smaller than − 23 kcal/mol (black line) are predicted to form β-sheet structures characteristic of amyloids, and are colored in red. In orange are the segments where Rosetta energies are between − 22 and − 23 kcal/mol, a unit larger than the threshold of − 23 kcal/mol. As with the sequence alignment, the aggregation profiles of these ubiquilin constructs differ mainly in the region between STI1-4 and the UBA domain. The UBQLN2 PXX repeat motif is not shown because none of its segments were predicted to form β-sheet structures. Asterisks designate the gaps in the multiple sequence alignment of the ubiquilins.

We previously identified the UBA domain as a key driver of UBQLN2 aggregation^13^. The UBA domains of all three brain-expressed ubiquilins are highly conserved, sharing 93% sequence identity (Fig. 1a)^30^. This similarity led us to investigate whether, like UBQLN2, UBQLN1 and UBQLN4 can also aggregate. Using an amyloid-like structure predictor algorithm^31^, we calculated the aggregation propensity profile of the three constructs used in our in vitro studies (Fig. 1b). These constructs (UBQLN1^438–589^, UBQLN2^430–624^ and UBQLN4^444–601^) extend from the fourth STI1 motif to the end of the protein and contain a 6xHis tag at the amino-terminus (Fig. 1). Whereas the UBA domains have nearly identical aggregation profiles, the fibril formation prediction profiles differ for the three constructs in the region between the fourth STI1 motif and the UBA domain (Fig. 1b). This region (fourth STI1 to UBA) also has the largest sequence variability across the ubiquilin constructs used for the in vitro studies and, in UBQLN2, is further distinguished by its unique proline-rich (PXX) domain.

Because the UBA domain is known to be critical for UBQLN2 aggregation and phase separation^13,19^, we anticipated that recombinant UBQLN1^438–589^ and UBQLN4^444–601^ would similarly aggregate in vitro*.* To measure aggregation, we used ThioflavinT (ThT), a dye that produces characteristic fluorescence in the presence of β-sheet structures and has been used to study fibril formation of many amyloid proteins^32,33^.

**Figure 2:**
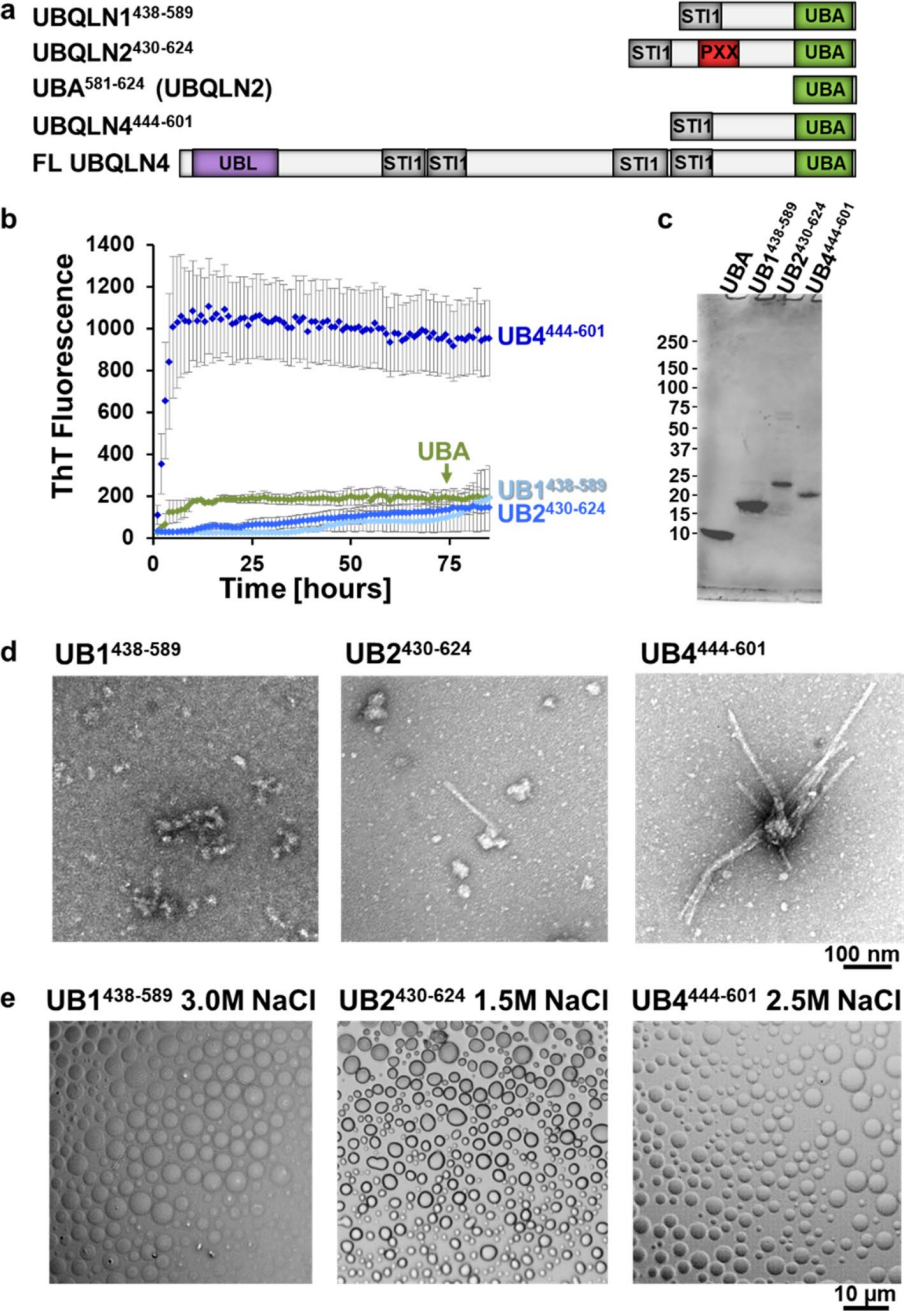
UBQLN4^444–601^ displays greater propensity to form amyloid-like aggregates than UBQLN1^438–589^ or UBQLN2^430–624^. (**a**) Constructs used for the studies of protein aggregation in vitro. The UBA^581–624^, a known driver of UBQLN2 aggregation, was tested as a positive control. FL UBQLN4 is shown for comparison at the bottom. (**b**) ThT assay was used to monitor aggregation of 10 µM ubiquilin constructs. The fluorescence is the mean of four or more independent experiments (bars = SEM). (**c**) SDS-PAGE of the recombinant proteins used in the ThT assays. (**d**) Samples for TEM imaging were collected at the termination of ThT assays. UBQLN4^444–601^ samples produced fibrillar species resembling amyloid fibrils and UBQLN2^430–624^ formed a heterogeneous mixture of small fibrils and globular species, while no fibrils were observed for UBQLN1^438–589^. (**e**) All ubiquilin constructs phase separated at 10 µM concentration. Images were taken with SteREO Discovery V20 (Zeiss) using visible light. Results were reproduced by two independent experiments.

Among the three ubiquilins, UBQLN4^444–601^ aggregated most rapidly (Fig. 2). At a 10 µM protein concentration, the UBQLN4^444–601^ aggregation reaction was the fastest to transition to a steady-state, characterized by a constant fluorescence over time. Indeed, the time to half transition to steady-state for UBQLN4^444–601^ was similar to that of the isolated UBA domain from UBQLN2, which drives aggregation of the full protein^13,19^. In contrast, UBQLN1^438–589^ and UBQLN2^430–624^ aggregated more slowly and displayed decreased ThT signal (Fig. 2b).

We note that for this study we used a UBA construct (UBA^581–624^) that is seven residues shorter than UBA^574–624^, which we previously described^13^. UBA^581–624^ lacks the thrombin cleavage site and a linker sequence present in the longer UBA^574–624^ construct^13^. These differences likely explain the shortened lag phase of UBA^581–624^ compared to UBA^574-624^ ^13^.

At reaction completion, aggregated samples were viewed by transmission electron microscopy (TEM). UBQLN4^444–601^ readily formed fibrillar aggregates resembling amyloid fibrils. In addition to the amyloid-like aggregates, we also observed some spherical UBQLN4^444–601^ species. In contrast, UBQLN1^438–589^ and UBQLN2^430–624^ mainly formed spherical species, though samples of UBQLN2^430–624^ contained low levels of sparsely distributed short fibrils (Fig. 2d). UBQLN4^444–601^ amyloid-like fibril formation likely explains its increased ThT fluorescence at steady-state, as ThT binds β-sheet structures characteristic of amyloids.

A previous study revealed that similarly truncated UBQLN2 constructs formed liquid-like condensates in solutions with higher ionic strength than the physiologically relevant PBS buffers^19^. This observation prompted us to compare the liquid–liquid phase separation potential of UBQLN1^438–589^ and UBQLN4^444–601^ to that of UBQLN2^430–624^. In our liquid–liquid phase separation experiments (Fig. 2e), we opted to use 10 µM as the starting protein concentration, which matched the protein concentrations of our aggregation assays, and screened buffers with increasing salt concentrations. The liquid–liquid phase separation assays, reproduced twice, were carried out with protein samples unlabeled with fluorescent tags. These data show that all ubiquilins liquid–liquid phase separate, but the images of UBQLN2^430–624^ samples differed from the ones of UBQLN1^438–589^ and UBQLN4^444–601^. We also found that the minimum salt concentration for liquid–liquid phase separation differed among the constructs (≥ 3 M NaCl for UBQLN1^438–589^; ≥ 1.5 M NaCl for UBQLN2^430–624^, and ≥ 2.5 M NaCl for UBQLN4^444–601^).

### Among tested ubiquilins, UBQLN4 is most prone to partition into the PBS-insoluble fraction of cell lysates

**Figure 3:**
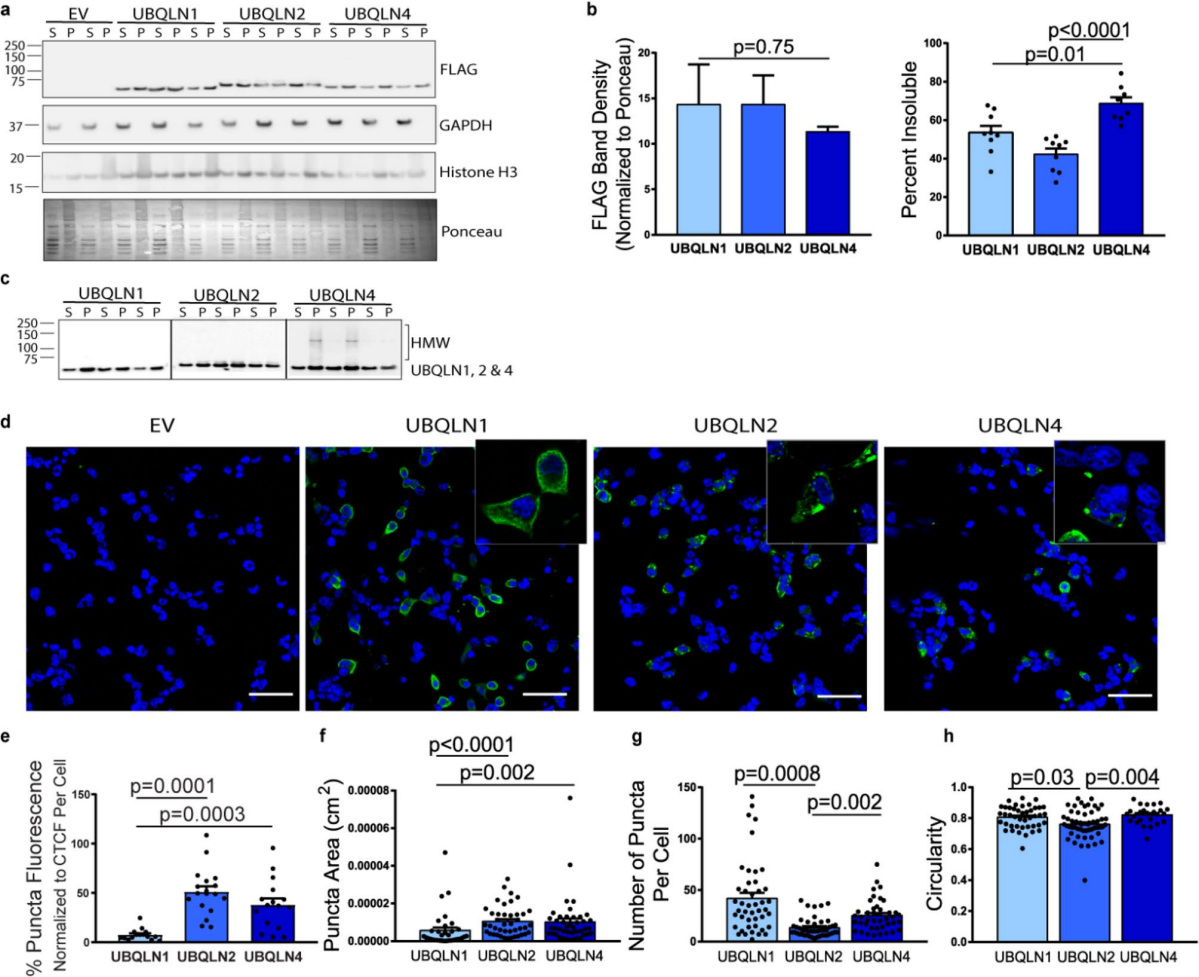
In transfected cells, UBQLN4 and UBQLN2 display enhanced aggregation and puncta formation compared to UBQLN1. (**a**) Of the three tested ubiquilins expressed in HEK-293 cells, FLAG-UBQLN4 is most prone to deposit in the PBS-insoluble (P) fraction versus the PBS-soluble (S) fraction (n = 9). Both cytoplasmic (GAPDH) and nuclear protein (Histone H3) is isolated in the soluble fraction. (**b**) All three ubiquilins were expressed equally as measured by total FLAG band density (normalized to Ponceau). For measurements of insolubility, values are expressed as percentage of protein that is PBS-insoluble, determined from the calculated total pool of PBS-soluble and PBS-insoluble. Data were analyzed by ANOVA. (**c**) UBQLN1, 2 and 4-specific antibodies (cropped to present all three antibodies in parallel) show that UBQLN4 partially electrophoreses as detergent-resistant high molecular weight species whereas UBQLNs 1 and 2 do not (uncropped Western blots are shown in Supplemental Figure S1). (**d**,**e**) Immunofluorescence of FLAG-UBQLNs 1, 2 and 4 shows that FLAG-UBQLN1 in HEK-293 cells is primarily diffusely expressed in the cytoplasm, whereas a higher percentage of FLAG-UBQLN2 and 4 is sequestered into large, heterogeneous puncta when normalized to corrected total cell fluorescence (CTCF) (n = 20). Characterization of puncta (n = 35–45/group) revealed that UBQLN1 puncta tend to be significantly smaller (**f**) and more numerous per cell (**g**) than UBQLN2 or UBQLN4 puncta**,** and UBQLN2 puncta are significantly less circular than either UBQLN1 or UBQLN4 puncta (**h**). Data were analyzed by Kruskal–Wallis test. Means and SEMs are displayed. Scale bar = 50 μm.

In our in vitro ThT and liquid–liquid phase separation assays we used truncated ubiquilin constructs that included the region of the protein known to drive phase separation and aggregation^13,19^. To determine whether the divergent aggregation and liquid-like behavior of ubiquilins observed in vitro extended to cells, we chose to analyze full-length proteins. We transiently expressed full-length FLAG-tagged UBQLNs 1, 2 and 4 (FLAG-UBQLN1, FLAG-UBQLN2, and FLAG-UBQLN4) in HEK-293 cells and separated cell lysates into PBS-soluble (supernatant) and PBS-insoluble (pellet) fractions. Because our mechanical shearing lysis protocol efficiently releases soluble proteins that normally reside in the nucleus (Fig. 3a), the vast majority of the proteins in the PBS-insoluble fraction are likely to be in an aggregated state as opposed to trapped in a subcellular structure. All three proteins partitioned into both PBS-soluble and insoluble fractions. Compared to FLAG-UBQLN1 and FLAG-UBQLN2, a significantly higher percentage of FLAG-UBQLN4 partitioned into the PBS-insoluble fraction (Fig. 3a,b). Moreover, on denaturing Western blots only FLAG-UBQLN4 was noted to electrophorese as a higher molecular weight, possibly dimeric species, and a high molecular weight (HMW) smear (Fig. 3c). This pattern of relative solubility/insolubility and formation of SDS-resistant HMW species by full-length ubiquilins mirrors the relative aggregation behavior of the C-terminal ubiquilin constructs in vitro.

To further evaluate assembly formation and aggregation of each ubiquilin, we quantified the formation of intracellular puncta by each protein in fixed cells. Assessed by immunofluorescence, FLAG-UBQLN1 primarily distributed diffusely in cells whereas a much greater percentage of FLAG-UBQLN2 and FLAG-UBQLN4 localized to cytoplasmic puncta (Fig. 3d,e). Characterization of puncta characteristics revealed that UBQLN1 puncta were smaller and more heterogeneous in number per cell than the larger puncta formed by both UBQLN2 and UBQLN4 (Fig. 3f,g). Analysis of the circularity of puncta revealed that UBQLN2 puncta were significantly less circular than either UBQLN1 or UBQLN4 puncta, which may be a function of their larger size and therefore physical constraint in the cellular space. To evaluate whether interactions between the three ubiquilins are necessary for the formation of UBQLN puncta in living cells, we assessed the formation of intracellular puncta by each protein in UBQLN1/2/4 triple knockout cells and found that, similar to control cells, all three UBQLNs form puncta in the absence of the other two ubiquilins (Supplemental Fig. S2).

### Divergent ubiquilin solubility in human brain

To gain insight into the physiological relevance of this divergence in ubiquilin aggregation, we analyzed levels of each brain-expressed ubiquilin in the PBS-soluble versus PBS-insoluble fractions of human brain lysates, assessing both age-matched controls and various neurodegenerative diseases.

**Figure 4:**
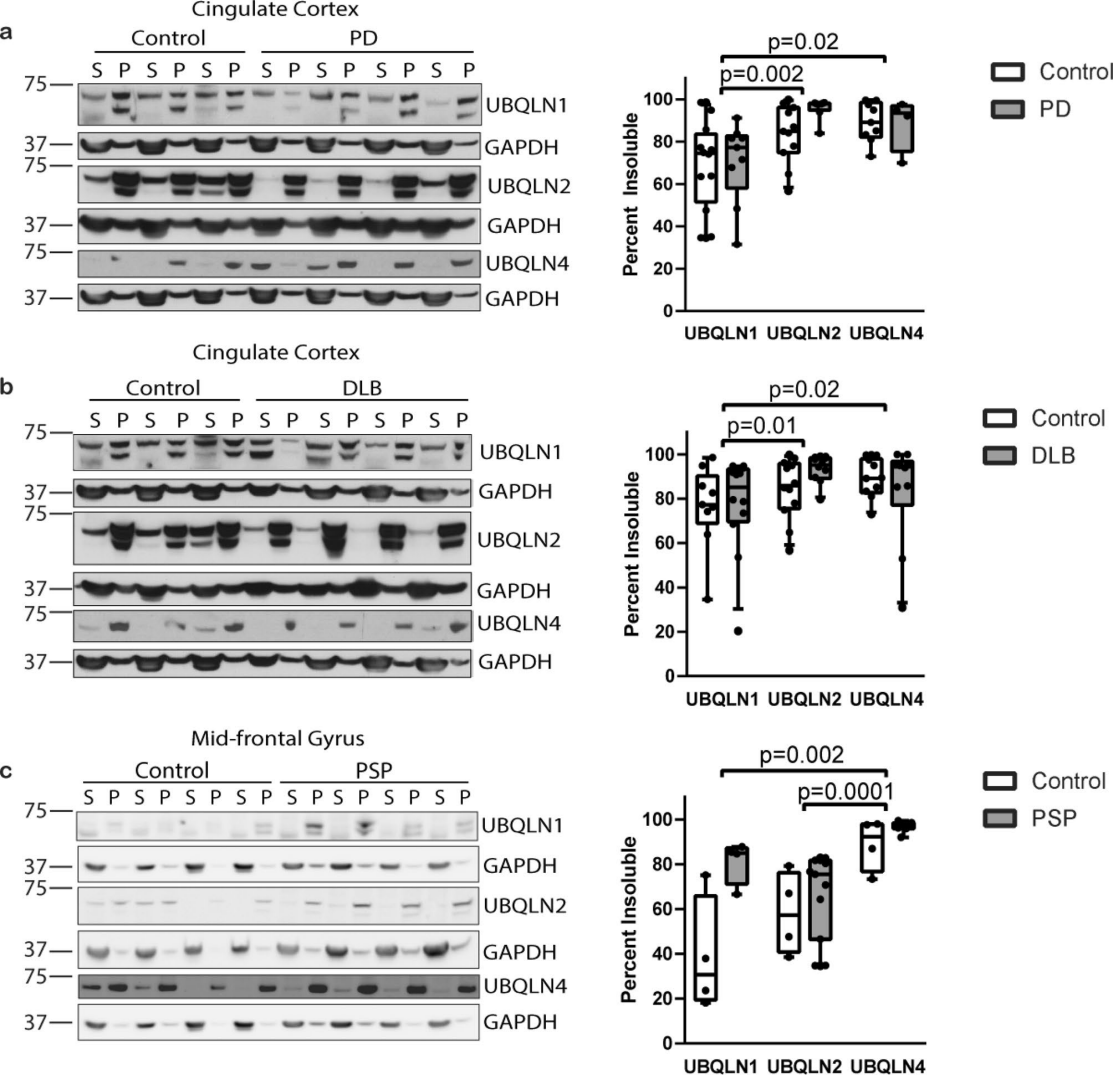
UBQLN2 and UBQLN4 are more insoluble than UBQLN1 in human brain tissue. Representative Western blots (uncropped Western blots shown in Supplemental Figure S3) of cingulate cortex from human controls (n = 9), PD (**a**; n = 6) and DLB (**b**; n = 15), and mid-frontal gyrus from human controls (n = 4) and PSP (**c**; n = 12). Results display increased partitioning of UBQLN4 into the insoluble fraction compared to UBQLN1, analyzed by the Kruskal–Wallis test grouping disease and controls together for each analysis. UBQLN2 insolubility is greater than UBQLN1 insolubility in both DLB and PD and respective controls, but significantly lower than UBQLN4 insolubility in PSP and respective controls. Results are expressed as a percentage of PBS-insoluble ubiquilin, determined from the calculated total PBS-insoluble (P) and PBS-soluble (S). No significant differences were seen between disease and controls for PD, DLB or PSP. Both UBQLN1 and UBQLN2 were detected and quantified as a doublet, as previously reported^13,52,53^. Data were analyzed by the Kruskal–Wallis test using the Dunn’s post-hoc test. Box-and-whisker plot (median, first and third percentiles, range of quantified bands) is displayed with the scatter plot of the raw data.

Results in human brain samples supported our results in vitro and in cellular models: the percentage of UBQLN4 present in the PBS-insoluble fraction was elevated over that of UBQLN1. The relative levels of UBQLN2 insolubility varied, but was intermediate between UBQLN1 and UBQLN4 (Fig. 4). We assessed cingulate cortex from two synucleinopathies, Parkinson’s disease (PD) and dementia with Lewy bodies (DLB), and frontal cortex from the tauopathy, progressive supranuclear palsy (PSP), as well as cortex from age-matched control brains (Supplemental Table S1). Comparisons of the soluble and insoluble fractions from both PD and DLB demonstrated that UBQLN2 and UBQLN4 behavior is similar, partitioning into the insoluble fraction to a greater extent than UBQLN1. Similarly, in PSP brain tissue, UBQLN4 insolubility was significantly elevated over UBQLN1 and UBQLN2. The inherent heterogeneity of human disease tissue samples likely explains the sample-to-sample variability in degree of solubility for the three ubiquilins. Notably, UBQLN4 displayed the least variation, consistently showing a high degree of insolubility in all three diseases, as well as in age-matched controls.

No disease-dependent differences in UBQLN4 insolubility were detected in PD (Fig. 4a), DLB (Fig. 4b) or PSP (Fig. 4c) when compared to age-matched control samples. Thus, both in control and disease brain tissue, UBQLN4, and to a lesser extent UBQLN2, preferentially exist in an insoluble form.

### Divergence in ubiquilin mobility within condensates assessed by recovery after photobleaching

Using fluorescence recovery after photobleaching (FRAP), we previously showed that UBQLN2 puncta in HEK-293T cells resemble liquid-like condensates and established a link between UBQLN2 condensate formation and aggregation^13^. The differential aggregation propensity of the three ubiquilins prompted us to compare the ubiquilins’ condensate mobility in cells. To assess mobility, we used wild-type human ubiquilins fused to eGFP and measured puncta FRAP in transfected cells. To reduce the chance that puncta exchanged contents with neighboring puncta, we examined their surroundings for other puncta using a Z-height scan. Data were recorded only from puncta that did not have immediately neighboring puncta and when the bleached area was 80% or smaller than the total punctum area. We bleached only the center of each punctum and used fixed ROI across all puncta, so that fluorescence recovery that did occur would likely take place from nonbleached regions of the same punctum.

**Figure 5:**
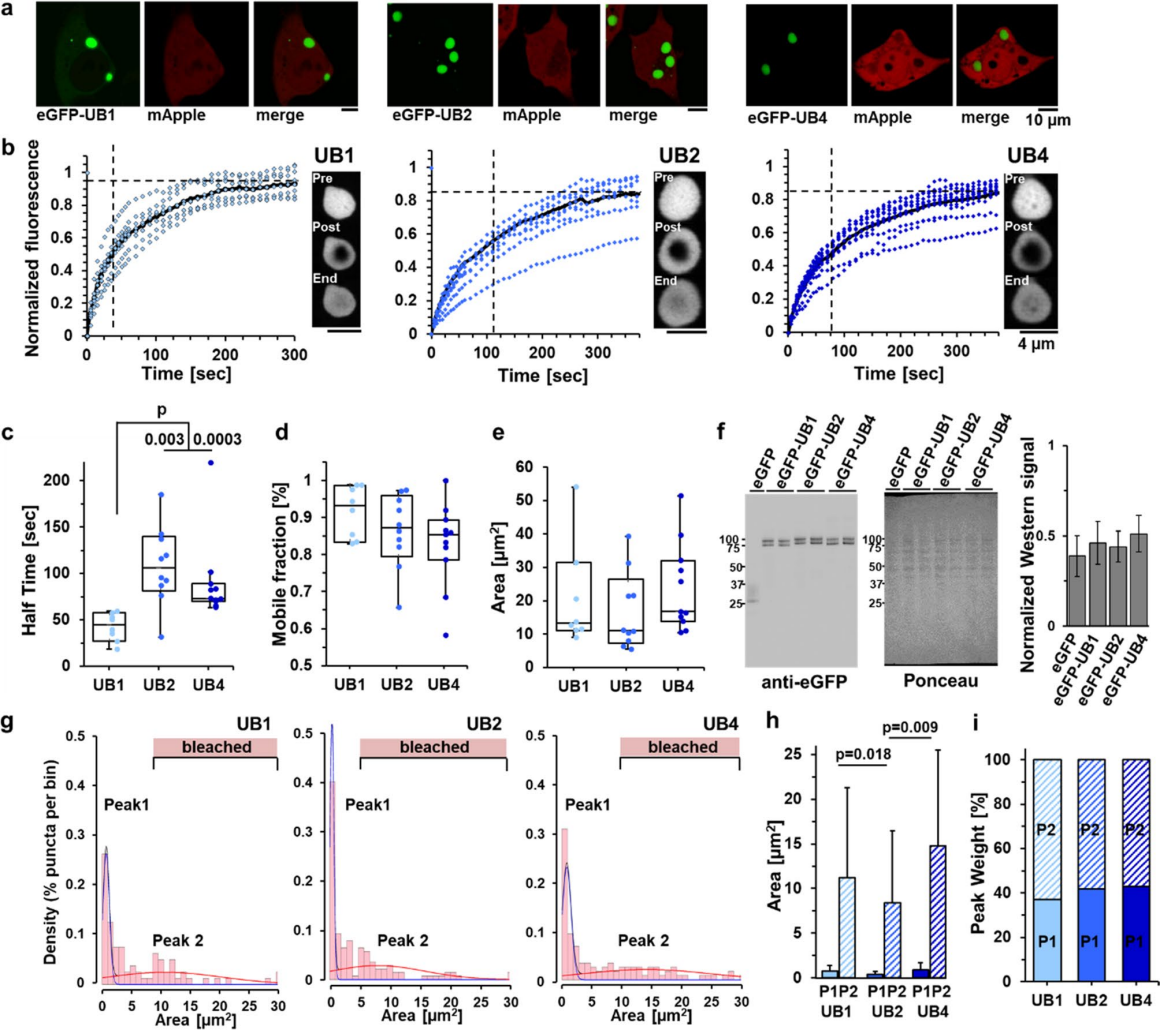
Dynamics of recovery after photobleaching of liquid-like ubiquilin condensates reveal divergent behavior of UBQLN2 and 4 versus UBQLN1. (**a**) Representative images of HEK-293 T cells transfected with eGFP-UBQLNs 1, 2 or 4 and mApple. (**b**) Black curves represent the mean fluorescence recovery of eGFP-UBQLNs 1, 2 and 4 puncta over time. FRAP of individual puncta is in blue. Dashed vertical and horizontal lines are half-time and mobile-fraction means, respectively. Representative images of puncta immediately before (top) and after (middle) photobleaching, and at the end of data collection (bottom) are shown to the right of each plot. (**c**) Time to 50% fluorescence recovery after photobleaching is slower for eGFP-UBQLN2 and eGFP-UBQLN4 than eGFP-UBQLN1. (**d**) FRAP measurements were used to compare protein mobility in puncta. All three brain-expressed ubiquilins show similar signal recovery (mobile fractions). Although not statistically significant, the mobile fractions for eGFP-UBQLN2 and 4 trended smaller than for eGFP-UBQLN1. (**e**) The area of puncta subjected to FRAP was similar for all three ubiquilins. (**f**) Representative Western blot (anti-eGFP antibody, left), and Ponceau stain (total protein loaded, middle), and quantification (right) of lysed HEK293T cells transfected with eGFP-ubiquilins confirm that eGFP-tagged ubiquilins were expressed at similar levels (means, SEM are displayed, N = 4). (**g**) Area distribution of puncta was modeled with two Gaussians defining two populations of puncta (maximum mixed model likelihood: − 251 for eGFP-UBQLN1, − 377 for UBQLN2, and − 375 for eGFP-UBQLN4). The pink bars labeled ‘bleached’ designate the size ranges of puncta selected for bleaching. (**h**) Population P1 contains uniformly small puncta, and population P2 contains the remaining puncta spanning a wide range of larger sizes. The punctum areas for eGFP-UBQLN1 and eGFP-UBQLN4 are larger than for eGFP-UBQLN2. (**i**) Percent distribution (population weight) of the three ubiquilins is similar, ranging from37-43% for P1, and 57–63% for P2. Data shown in (**g**–**i**) panels include puncta in the images taken from live HEK293-T cells transfected with eGFP-ubiquilins. Box-and-whisker plot (median, first and third percentiles, range of data for panels (**c**–**e**) display the scatter plot of the raw data (N = 5). Data were analyzed with the Kruskal–Wallis test^50^.

Consistent with the above results, eGFP-UBQLN1 fluorescence recovery was faster than that of UBQLN2 or UBQLN4 (Fig. 5c). To minimize any effect of puncta drift from the photobleached region (region of interest (ROI)), we chose shorter recording times of 300 s for UBQLN1 puncta, which were the fastest to recover fluorescence signal. Although the mobile fractions for all ubiquilins were similar, the mobile fractions for UBQLN2 and 4 trended lower than for UBQLN1 (Fig. 5d). Recovery times of UBQLN2 and 4 puncta are similar to one other and longer than those of UBQLN1 puncta (Fig. 5c). In addition to FRAP experiments, we recorded examples of puncta fusion by UBQLNs 1, 2, and 4 (Supplementary Movies 1–7). Supplementary Movie 8 shows an example of puncta fission by UBQLN4. Taken together, these results suggest that UBQLN2 and UBQLN4 display similar self-association in liquid-like condensates, differing from UBQLN1.

Modeling with normal (Gaussian) distributions revealed that the size of puncta formed by all three ubiquilins followed similar area distributions, consisting mainly of two populations (Fig. 5g–i). The first population, comprising 37–43% of puncta, was relatively small in size (mean areas: 0.8 ± 0.6 µm^2^ for eGFP-UBQLN1, 0.4 ± 0.3 µm^2^ for eGFP-UBQLN2, and 0.9 ± 0.8 µm^2^ for eGFP-UBQLN4), and the second population was much larger and distributed over a wider size range (mean areas: 11.2 ± 10.1 µm^2^ for eGFP-UBQLN1, 8.4 ± 8.1 µm^2^ for eGFP-UBQLN2, and 14.8 ± 10.7 µm^2^ for eGFP-UBQLN4). Linear modeling of punctum area in live cells overexpressing ubiquilin proteins showed that larger puncta tend to be less circular (Supplemental Table S2, Fig. 5g–i). Overall, punctum areas of eGFP-UBQLN1 and eGFP-UBQLN4 were larger for both the small and large populations compared to eGFP-UBQLN2. We note that while overall patterns of differences between UBQLN2 and 4 versus UBQLN1 are consistent between multiple systems we have used to analyze the three ubiquilins, the circularity and puncta area of eGFP-tagged proteins differs from that of FLAG-tagged proteins visualized in fixed cells. Differences between the size of the protein tags and/or the dynamic species measured in live-imaging versus in fixed cells may underlie differences. Puncta selected for FRAP all fall within the larger population of condensates (Fig. 5g–i).

## Discussion

All three brain-expressed ubiquilins are linked to neurodegenerative diseases characterized by protein dyshomeostasis^1–3,5,29^. Recent studies highlight UBQLN2 propensity to form amyloid-like fibrils and liquid-like condensates^13,19,20^, but the relative aggregation behavior of these three highly homologous proteins has not been investigated. Here we report that the three ubiquilins share the property of liquid–liquid phase separation (Figs. 2e, Fig. 5), yet differ in condensate properties (Fig. 5), propensity to form fibrillar aggregates (Fig. 2), and accumulation in insoluble species in cells and brain tissue (Figs. 3, 4).

Although all recombinantly-expressed ubiquilins phase separate, expanding upon previous study of recombinant UBQLN2 phase separation^19,20^, we observed that the minimum salt concentration for liquid–liquid phase separation differed among the constructs (≥ 3 M NaCl for UBQLN1^438–589^; ≥ 1.5 M NaCl for UBQLN2^430–624^, and ≥ 2.5 M NaCl for UBQLN4^444–601^). Because we are using unlabeled proteins in our in vitro assays, we cannot formally conclude that any specific ubiquilin protein has partitioned inside or outside of the liquid droplets. Our results suggest that similar to our studies with mammalian cells, all recombinantly expressed ubiquilins have the capacity to phase separate under a specific set of conditions.

With respect to the shared property of liquid–liquid phase transition, UBQLN1 is most liquid-like and UBQLN4 most aggregation-prone among the three tested ubiquilins in mammalian cellular systems overexpressing full-length proteins (Fig. 5). Previous studies have suggested that liquid droplets formed by aggregation-prone proteins proceed to a less liquid state (from hydrogel to amyloid) at a faster rate^34^. Consistent with our earlier findings with UBQLN2 and studies by others of additional aggregation-prone proteins^13,15,17,35^, the increased recovery times after photobleaching for UBQLN4 puncta (Fig. 5) correlate with its tendency to form high molecular weight aggregates and partition into the insoluble fraction (Figs. 3, 4). Some studies have shown that amyloid fibrils can induce liquid–liquid phase transition, implying that the relationship could be bi-directional^36,37^. The correlation between increased recovery times of UBQLN4 liquid-like droplets and heightened fibril formation suggests that regulation of liquid–liquid phase transition by ubiquilins is linked to aggregation.

The ability of all ubiquilins to form liquid-like droplets suggests it is a fundamental shared characteristic of this class of proteins that relates, in a still undefined manner, to their function in quality control pathways. While the relevance of liquid-like droplet formation to ubiquilin function is not yet understood, studies of other protein quality control factors may provide insight into potential mechanisms. For example, proteasome components form liquid-like droplets that depend on the interaction of polyubiquitin chains with the UBA–UBL ubiquitin–proteasome shuttle factor, Rad23B^38^. Thus, ubiquilins may form concentrated molecular assemblies via their UBL and UBA domains to regulate multiple cellular processes.

Despite being highly similar, the brain-expressed ubiquilins differ in condensate and aggregation behavior. These differences suggest that relatively minor sequence changes can alter the protein’s behavior. In this light, one can envision that missense mutations that cause UBQLN2-mediated disease^1^, or possibly UBQLN4-mediated disease^5^, would change these proteins’ behavior along the liquid-like droplet-hydrogel-aggregate spectrum. Published evidence from our group and others supports this view^13,19,20^. While the ubiquilins’ UBL and UBA domains are nearly identical, the three brain-expressed UBQLNs are distinguished by the middle region located between the UBL and UBA domains^30^. In evaluating the C-terminal regions of UBQLNs 1, 2 and 4 for fibril formation, we demonstrated that UBQLN4^444–601^ was the most aggregation-prone, while UBQLN1^438–589^ and UBQLN2^430–624^ displayed slower aggregation kinetics. These results suggest that C-terminal differences among brain-expressed ubiquilins lead to altered aggregation behavior that corresponds to the differences observed for the full-length proteins.

Although UBQLN4 exhibited the greatest propensity to aggregate in in vitro assays, cellular models, and human brain tissue, UBQLN2 also displayed heightened aggregation propensity in cellular models and human brain when compared to UBQLN1. Since UBQLN2 is known to accumulate in aggregate-like structures in various neurodegenerative diseases^1, 3,29,39^, it will be important now to determine whether UBQLN4 similarly accumulates in one or more neurodegenerative diseases.

Liquid-like condensates are complex structures with antagonistic behaviors. While many important cellular functions are carried out in liquid-like condensates, phase separation has been implicated in abnormal protein aggregation and neurodegeneration^22–25^. Interestingly, it has been observed that UBQLN2 can both protect and facilitate abnormal protein aggregation in the context of liquid–liquid phase separation. Indeed, wild-type UBQLN2 protects against pathogenic protein aggregation in FUS-related ALS/FTD by decreasing stress granule formation^26^. In contrast, liquid-like condensates of UBQLN2 may facilitate TDP43 aggregation in ALS/FTD^21^. Although UBQLN4’s involvement in protein aggregation is yet to be determined, it is possible that UBQLN4 liquid-like condensates, like UBQLN2, are sites that initiate and facilitate abnormal protein association.

Our results demonstrating that UBQLN4 insolubility is elevated over UBQLN1—and in some cases over UBQLN2—in human brain suggest that heightened UBQLN4 aggregation behavior may be an intrinsic feature of the protein that informs its function. Furthermore, the percent UBQLN4 in the insoluble fraction showed little variability between samples despite the inherent wide variation present when using human tissue. While no differences in UBQLN4 solubility were detected in PD, DLB or PSP samples compared to age-matched controls, our limited analysis of available human samples does not allow us to conclude that there are no disease-dependent changes. Further studies are needed to elucidate whether UBQLN4 aggregation status relates to specific diseases or more generally to brain aging.

Although the relationship between dynamic molecular assembly formation and ubiquilin function is still uncertain, we anticipate that the phase separation and aggregation behavior of all three brain-expressed ubiquilins will prove to be regulated by ubiquitin and will inform their function as proteostasis regulatory factors^19^. Our results reveal two populations of condensates formed by all three ubiquilins, one being uniformly small and the second being larger and more variable in size. Small condensates conceivably represent functional assemblies, whereas larger condensates do not. Studies have also shown that the three ubiquilins interact with one another, indicating that their cellular functions and liquid–liquid phase transition properties may be linked^40^, though we show that all three ubiquilins form puncta in cells absent the presence of inter-ubiquilin interactions. Further research will be needed to better understand how the shared and divergent condensate and aggregation properties of ubiquilins influence their cellular functions and roles in disease.

## Methods

### Plasmids

The pCMV4-FLAG-UBQLN2 plasmid (p4455 FLAG-hPLIC-2; Addgene plasmid # 8661) and pCS2-FLAG-UBQLN1 plasmid (p4458 FLAG-hPLIC-1; Addgene plasmid # 8663) were gifts from Peter Howley^41^. UBQLN4 was cloned from pDONR223-UBQLN4 (pENTR-A1UP; Addgene plasmid # 16170), which was a gift from Huda Zoghbi (Baylor College of Medicine), into the pCMV4-FLAG vector. Control empty vector plasmid for FLAG cell transfection experiments was pCMV-HA. eGFP-UBQLN1, 2 and 4 were cloned from FLAG-tagged plasmids and eGFP. The pRP[Exp]-Neo-CMV-eGFP, pRP[Exp]-Neo-CMV-eGFP-UBQLN1, pRP[Exp]-Neo-CMV-eGFP-UBQLN2, and pRP[Exp]-Neo-CMV-eGFP-UBQLN4 were purchased from VectorBuilder. The plasmids for bacterial expression of ubiquilin constructs (pET3a-His6-W-UBQLN1^438–589^, pET3a-His6-W-UBQLN2^430–624^, pET3a-His6-W-UBQLN4^444–601^ pET3a-His6-W-UBQLN2^581–624^(UBA)) were purchased from Genscript.

### Protein expression and purification

All plasmids for bacterial expression contained a 6xHis tag and a tryptophan (Trp) at the N-terminus. UBQLN1^438–589^, UBQLN2^430–624^ and UBQLN4^444–601^ constructs were transformed in Rosetta (DE3) Escherichia coli bacteria. All LB agar plates and LB media were supplemented with 100 μg/mL carbenicillin and 34 μg/mL chloramphenicol. Transformed cells were grown overnight at 37 °C on LB agar media plates. The following day, cells were transferred to 100 mL LB starter cultures and allowed to grow for 60 min, then upscaled into 1 L LB media. At OD601 ≈ 0.6–0.8, cells were induced with 0.5 mM isopropyl β-d-1-thio-galacto-pyranoside (IPTG) and collected after 4 additional hours of incubation. Bacteria were collected by centrifugation for 10 min at 10,322×g. Bacterial pellets were stored at − 80 °C.

For protein purification, pellets of bacteria grown in 1 L of LB media were resuspended in 25 mL of pre-chilled lysis buffer containing 2% glycerol, 1 mM EDTA, 25 mM Na phosphate, pH 7.4, EDTA-free cOmplete protease inhibitor cocktail tablet (Roche; 1 tablet per 10 mL lysis buffer) and 6 μL/mL of saturated phenylmethylsulfonyl fluoride (PMSF). Bacteria were then lysed using EmulsiFlex B-15 high pressure homogenizer (Avestin). The lysates were centrifuged at 31,000×g for 20 min and the supernatant was added to Ni-NTA agarose slurry, After incubation for 15 min at 4 °C, the slurry was washed with 25 mM Na phosphate pH 7.4 containing 0.1 M NaCl, 6 μL/mL from saturated PMSF 2% glycerol, and 20 mM imidazole. Proteins were eluted with 25 mM Na phosphate pH 7.4, which was supplemented with 200 mM imidazole 0.1 M NaCl, 6 μL/mL from saturated PMSF, and 2% glycerol. In the case of the UBQLN2^430–624^ construct, the eluted protein was diluted with two volumes of 25 mM Na phosphate pH 7.4 containing 2% glycerol. Then it was filtered through 50 kDa concentrator (Amicon Ultra-15 Centrifugal Filter Unit, Millipore Sigma) and the flow through was collected for dialysis. Proteins were dialyzed against 5 mM Na phosphate pH 7.4 with two buffer exchanges overnight. Immediately after dialysis, proteins were frozen in liquid nitrogen and stored at − 80 °C. Protein concentration was determined by Pierce BCA protein assay Kit (cat. # 23225, ThermoFisher Scientific).

### ThioflavinT binding assay

ThT assays were carried out with 10 μM protein (UBQLN1^438–589^, UBQLN2^430–624^ and UBQLN4^444–601^) in 0.1 M NaCl, 1 mM sodium azide, and 20 mM Na phosphate pH 7.5. Prior to the assay, proteins were filtered through a 0.22 μm filter. ThT was added to a final concentration of 10 μM. Teflon beads were added into each well of a Falcon 96-well plate (black/clear, flat bottom, Corning, cat. # 353219). Then 75 μL of sample were pipetted into each well. Plates were then incubated at 37 °C in a FLUOstar Omega (BMG Labtech Inc) by shaking at 200 rpm using the ‘meander corner well shaking’ mode. Fluorescence was measured with gain set at 90%, an excitation wavelength of 440 nm and emission wavelength of 490 nm. Three technical replicates were measured per sample for a single ThT assay. Data shown in Fig. 1 are averaged over three to four independent experiments done with different protein preparations.

### Transmission electron microscopy (TEM)

Negatively stained specimens for TEM were prepared by applying 5 μL of protein (UBQLN1^438–589^, UBQLN2^430–624^ and UBQLN4^444–601^) sample to hydrophilic 400 mesh carbon-coated Formvar support films mounted on copper grids (Ted Pella, 01702-F). Samples were allowed to adhere for 4 min, rinsed twice with distilled water, and stained for 60–90 s with 5 μL of 1% uranyl acetate (Ted Pella, Inc.). All samples were imaged at an accelerating voltage of 80 kV in a JEOL JSM 1400 Plus (JOEL).

### Liquid–liquid phase separation assays

Liquid–liquid phase separation assays were carried out using hanging-drop vapor diffusion. 2 μL of 10 μM protein (UBQLN1^438–589^, UBQLN2^430–624^ and UBQLN4^444–601^) in 5 mM Na phosphate pH 7.5 were mixed with 1 μL of reservoir solution. The drop was equilibrated over 1 mL of reservoir solution at 37 °C. The reservoir solutions that were tested contained 1.0 M, 1.5 M, 2.0 M, 2.5 M, 3.0, and 3.5 M NaCl in 1 mM EDTA, 1 mM sodium azide, and 25 mM Na phosphate pH 7.5. Samples were imaged with SteREO Discovery V20 (Zeiss) at 2 days and 14 days. Data were reproduced twice.

### HEK cell transfection

Human embryonic kidney 293 (HEK-293; Batch # 70008735, ATCC) cells were cultured in high glucose DMEM, supplemented with 10% FBS, 10 mM Glutamine and 100 U/mL penicillin/streptomycin. UBQLN1, 2 and 4 triple knockout HEK-293 cells^42^ were cultured in high glucose DMEM, supplemented with 10% FBS, 10 mM Glutamine, 10 µg/mL blasticidin and 100 µg/mL hygromycin. Cells were transfected with either pCMV-FLAG-UBQLN1, pCMV4-FLAG-UBQLN2, pCMV4-FLAG-UBQLN4 or pCMV-HA using Lipofectamine-2000 according to the manufacturer’s instructions.

For FRAP experiments, HEK-293T (cat. # CRL 3216, ATCC Batch No. 70008735) cultured in DMEM (cat. # SH30242.01 ThermoFisher Scientific), 10% FBS and 100 U/mL penicillin/streptomycin, were co-transfected with constructs of eGFP fusion proteins (proteins as specified), and mApple (for visualization of cells).

### Human disease brain tissue

Frozen brain tissue from the cingulate cortex was obtained from subjects with PD, DLB, and age-matched control subjects as well as mid-frontal cortex from PSP and age-matched control subjects (Supplemental Table S1) from the Michigan Brain Bank (University of Michigan, Ann Arbor, MI, USA). Brain tissue was collected with the informed consent of the patients. Protocols were approved by the Institutional Review Board of the University of Michigan and abide by the Declaration of Helsinki principles. Samples were examined at autopsy by neuropathologists for diagnosis.

### Western blot analysis

Cell lysates were homogenized using 0.2 mm stainless steel beads, sonicated for 5 min in chilled water, centrifuged at 10,000 rcf for 10 min at 4 °C and supernatants were collected. For insoluble fractions, pellets were resuspended in PBS with protease inhibitor cocktail (catalog no. 11873580001; Sigma Aldrich), centrifuged at 10,000 rcf for 10 min at 4 °C, and supernatants were discarded. Remaining pellet was resuspended in 1% sarkosyl in PBS with protease inhibitor, vortexed for 1 min, and incubated at room temperature for 30 min. Samples were water sonicated for 5 min and centrifuged for 20 min at 14,000 rpm at 4 °C. Protein concentrations were measured by BCA (cat. # 23227, ThermoScientific). Cell lysates containing 10 μg of total protein were loaded (without boiling) on precast NuPAGE 4–12% Bis–Tris gels (Invitrogen) for SDS-PAGE analysis. Gels were subsequently transferred onto nitrocellulose membranes and stained with Ponceau-S for total protein quantification. After destaining, membranes were blocked for 1 h at room temperature with 10% nonfat dry milk in TBS-T buffer. Membranes were then probed overnight at 4 °C in anti-FLAG, clone M2 (Sigma, cat. # F3165; 1:1000), anti-Ubiquilin-2 (Novus Biologicals, cat. # NBP2-25164; 1:2000), anti-Ubiquilin-1 (Novus biologicals, cat. # H00029979-M02; 1:2000), anti-A1UP (Santa Cruz, cat. # sc-136145; 1:2000), anti-Histone H3 (Cell Signaling, cat. # 4499; 1:2000) or anti-GAPDH (Millipore, cat. # MAB374; 1:5000) diluted in 5% nonfat dry milk. HRP-conjugated goat anti-rabbit IgG or goat anti-mouse IgG (1:5000; ThermoFisher Scientific, cat. # 31460 and 32430) were used for detection as appropriate. All ubiquilin antibodies were tested without stripping. In replicate Western blots, secondary antibodies were inactivated by incubating with H_2_O_2_ for 15 min at 37 °C before adding another antibody of a different species. ECL (Pierce) was used to visualize bands using the Gbox Mini-6 (Syngene), which were normalized to corresponding total protein levels detected by Ponceau-S. All quantification of immunoblots was performed by densitometric analysis using Genetools software (Syngene). Experiments were completed in triplicate and analyzed by one-way ANOVA with the Bonferroni post-hoc analysis. Data were analyzed using Statview (SAS Institute).

HEK-293T cells (cat. # CRL 3216, ATCC Batch No.70008735) transfected with eGFP-tagged ubiquilins were resuspended in ice cold PBS 0.5% Triton-X 100 with cOmplete, Mini, EDTA-free Protease Inhibitor Cocktail (cat. # 11836170001, Millipore Sigma/Roche), and sonicated in Bioruptor Pico (Diagenode) using 4 sonication cycles (each 30 s ON/30 s OFF) at 4 °C. After centrifugation at 10,000 rcf for 10 min at 4 °C, supernatants were snap frozen in liquid nitrogen and stored at − 80 °C until use. Pierce BCA protein assay Kit (cat. # 23225, ThermoFisher Scientific). Samples containing 5.5 μg of total protein supplemented with NuPage LDS sample buffer (cat. # NP0007, ThermoFisher Scientific) were loaded without boiling on NuPAGE 4–12% Bis–Tris gels (cat. # WG1403BOX, ThermoFisher Scientific) with 1 × NuPage MES SDS Running Buffer (cat. # NP0002, ThermoFisher Scientific) for SDS-PAGE analysis. Gels were subsequently transferred onto nitrocellulose membranes (0.45 µm, cat. # 162-0115, BioRad) for 1 h at 110 V and stained with Ponceau-S for total protein quantification. After destaining, membranes were blocked for 1 h at room temperature with 10% Applichem nonfat dried milk (cat. # NC0167677, ThermoFisher Scientific) in TBS-T buffer. Membranes were then probed overnight at 4 °C in anti-GFP (mouse monoclonal IgG1κ, cat. #: 11814460001 Millipore Sigma/Roche) diluted 1:1000 in 5% Applichem nonfat dried milk in TBS-T. HRP-conjugated goat anti-mouse IgG (diluted 1:3000 in 5% Applichem nonfat dried milk in TBS-T) were used for detection. Advansta WesternBright ECL HRP Substrate Kits (cat. # 490005-020, WVR) were used to visualize protein bands. Protein bands of five independent experiments were quantified using ImageJ^43^ and normalized for the total protein amount detected by Ponceau-S.

Soluble and insoluble human brain samples were homogenized in PBS with a protease inhibitor cocktail (catalog no. 11873580001; Sigma Aldrich) with 3.2 mm stainless steel beads, using a 1:3 dilution of tissue: PBS (w/v). Samples were centrifuged at 10,000 rcf for 10 min at 4 °C. Supernatants (PBS-soluble fraction) were aliquoted, snap-frozen, and stored at − 80 °C until use. For insoluble fractions, pellets were resuspended in PBS with the protease inhibitor cocktail, centrifuged at 10,000 rcf for 10 min at 4 °C and supernatants were discarded. The remaining pellet was resuspended in 1% sarkosyl in PBS with protease inhibitor, vortexed for 1 min, and incubated at room temperature for 1 h. Samples were water sonicated for 5 min and centrifuged for 20 min at 14,000 rpm at 4 °C. Supernatants were discarded and procedure was repeated with remaining pellet for insoluble fraction. Protein concentrations were measured by BCA (cat. # 23227, ThermoScientific). Brain extracts containing 25 μg of total protein were loaded (without boiling) on precast NuPAGE 4–12% Bis–Tris gels (Invitrogen) for SDS-PAGE analysis. Gels were subsequently transferred onto nitrocellulose membranes and blocked for 1 h at room temperature with 10% nonfat dry milk in TBS-T buffer. Membranes were then probed overnight at 4 °C in anti-Ubiquilin-2 (Novus Biologicals, cat. # NBP2-25164; 1:2000), anti-Ubiquilin-1 (Novus biologicals, cat. # H00029979-M02; 1:2000), anti-A1UP for UBQLN4 (Santa Cruz, cat. # sc-136145; 1:2000), or anti-GAPDH (Millipore, cat. # MAB374; 1:5000) diluted in 5% nonfat dry milk. HRP-conjugated goat anti-rabbit IgG or goat anti-mouse IgG (1:5000; ThermoFisher Scientific, cat. # 31460 and 32430) were used for detection as appropriate. ECL (Pierce) was used to visualize bands using the Gbox Mini-6 (Syngene), which were normalized to corresponding GAPDH levels. All quantification of immunoblots was performed by densitometric analysis using ImageJ software (National Institutes of Health)^43^.

To quantify the percent insoluble protein for each sample, normalized insoluble ubiquilin band densities were corrected for ubiquilin concentration based on lysate volume, and then divided by total (soluble + insoluble) normalized ubiquilin protein levels and multiplied by 100. Two-way ANOVA revealed UBQLN-dependent differences, but no differences based on disease state. Therefore, control and disease samples were pooled for one-way ANOVA analyses of differences in UBQLN insolubility. Analyses were completed in triplicate and analyzed by the Kruskal–Wallis test with the Dunn’s post-hoc test. Data were analyzed using Statview (SAS Institute).

### Immunofluorescence

Fixed cells were washed in PBS, permeabilized with 0.5% Triton-X 100 and blocked in 5% goat serum for 1 h. Cells were incubated in anti-FLAG, clone M2 (Sigma cat. # F3165, 1:100) overnight at 4^ο^C. The following day, cells were washed in PBS three times for 10 min each and incubated with goat anti-mouse IgG Alexa-568 (Invitrogen cat. # A-11004; 1:500) for 1 h. Sections were then washed in PBS three times for 10 min each and incubated with DAPI (Sigma) to label nuclei for 5 min at room temperature, washed three times for 5 min each, and were mounted with Prolong Gold Antifade Reagent (Invitrogen). Slides were imaged using an IX71 Olympus inverted microscope. Images were analyzed with the Analyze Particles tool in Image-J (National Institute of Health)^43^ to determine the corrected total cellular fluorescence (CTCF; used as a measure of overall expression of each ubiquilin protein per cell for normalization), the corrected total fluorescence for each punctum (CTFP), circularity and area of each punctum and total number of puncta per cell. To calculate the percent fluorescence of puncta, the sum of CTFP of all puncta in the cell was divided by CTCF of the whole cell. Immunofluorescence experiments were repeated in triplicate and statistical analyses utilized means from the three replicates. Analyses were completed by the Kruskal–Wallis test and Dunn’s post-hoc test. Data were analyzed using Statview (SAS Institute).

### Fluorescence recovery after photobleaching (FRAP)

HEK293-T cells (Batch # 70008735, ATCC) were plated on Nunc Lab-Tek II Chambered Coverglass (cat. # 155409, Fisher) in DMEM, supplemented with 10% FBS, 10 mM Glutamine and 100 U/mL penicillin/streptomycin. The next day cells were transfected with eGFP-UBQLN1, eGFP-UBQLN2, and eGFP-UBQLN4 plasmid DNA. Using Lipofectamine 2000 Transfection Reagent (cat. # 11668027; Fisher) according to the manufacturer’s instructions. Cells were imaged 24 h after transfection with a Nikon A-1 confocal microscope (40 × WI Lens) using Nikon Elements software with perfect focus engaged. For FRAP data collection, an area of 512 pixels was scanned using speed 518 frames/sec. FRAP imaging consisted of three phases: pre-bleach, bleaching, and post-bleach imaging. During pre-bleach imaging, each punctum was imaged every 2 s. In the bleach phase, a 5–6 μm^2^ ROI was drawn in the middle of the punctum covering no more than 2/3. This ROI defined the stimulation area for a 488 nm laser with 20% power. The post-bleach phase consisted of two periods. For the first minute, images were acquired every 5 s, while for the subsequent 6 min, images were acquired every 10 s. This was done to provide greater temporal resolution during the rising phase of fluorescence recovery. FRAP analysis was performed in Fiji^44^. To fix granules in place, stack registration (Rigid Body) was performed. Following thresholding, one ROI was generated that corresponded to the pre-bleach signal and another to the post-bleach signal. A mask was created from the pre-bleach ROI, inverted, and the post-bleach ROI was subtracted from this image. This created a region of bleached signal deemed the FRAP ROI. At each time point the integrated density of the FRAP ROI was divided by the integrated density of the pre-bleach ROI. These values were then normalized such that the mean of the five pre-bleach values was set to 1, and the first postbleach value was set to 0. To calculate the mobile fraction and half time, the normalized fluorescent signal measured from each granule was fitted to the equation A * (1 − exp(− t/τ)), where A is the mobile fraction, t is the collection time and τ is the half time^45^. Puncta area, circularity, roundedness, aspect ratio. and solidity were calculated using Fiji^44^.

The area distribution of UBQLN1, 2, and 4 puncta was modeled using Normal (μ, σ2) distribution. The process of distribution model-fitting involved maximum likelihood estimation of the model distribution parameters^46^. We employed the R packages mixtools^47^ and ggplot2^48^ to obtain and plot the desired models. The model parameters and the mixture distribution weights are used as proxy measures to discriminate between different puncta areas. For statistical analysis of FRAP, Kruskal–Wallis (KW) test was used, which is an alternative when parametric assumptions are not guaranteed^49,50^. FRAP data are shown as means ± SEM.

## Supplementary Information

Supplementary Information.

Supplementary Video 1.

Supplementary Video 2.

Supplementary Video 3.

Supplementary Video 4.

Supplementary Video 5.

Supplementary Video 6.

Supplementary Video 7.

Supplementary Video 8.
