# Supplementary material for: Shared and divergent phase separation and aggregation properties of brain-expressed ubiquilins
Source: Sci Rep. 2021 Jan 11;11:287. doi: 10.1038/s41598-020-78775-4 (PMC7801659; doi:10.1038/s41598-020-78775-4)
Supplement: Supplementary file 1 — Supplementary Information. [file 41598_2020_78775_MOESM1_ESM.pdf]

Supplemental Information

Title: Shared and divergent phase separation and aggregation properties of brain-expressed ubiquilins

Authors: Julia E. Gerson, Hunter Linton, Jiazheng Xing, Alexandra B. Sutter, Fayth S. Kakos, Jaimie Ryou, Nyjerus Liggans, Lisa M. Sharkey, Nathaniel Safren, Henry L. Paulson, Magdalena I. Ivanova

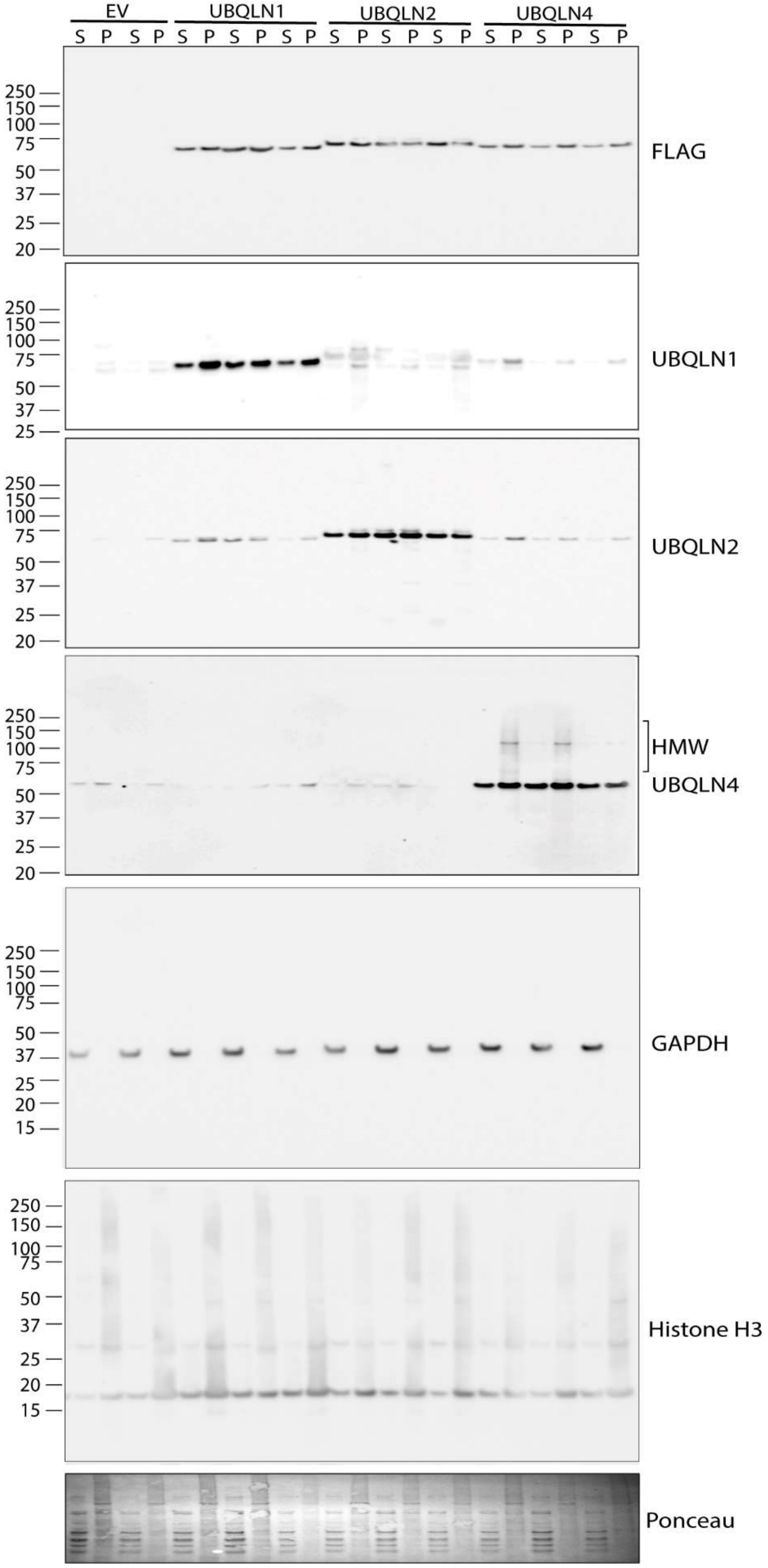

EV
UBQLN1
UBQLN2
UBQLN4
S P S P S P S P S P S P S P S P S P S P S P S P
250
150
100
75
50
37
25
20
15
FLAG
UBQLN1
UBQLN2
HMW
UBQLN4
GAPDH
Histone H3
Ponceau

Supplementary Figure S1.

Full, uncropped Western blots of cell lysates from Figure 3 detected with UBQLN1, 2 and 4-specific antibodies demonstrate specificity of each antibody in measuring the ubiquilin proteins.

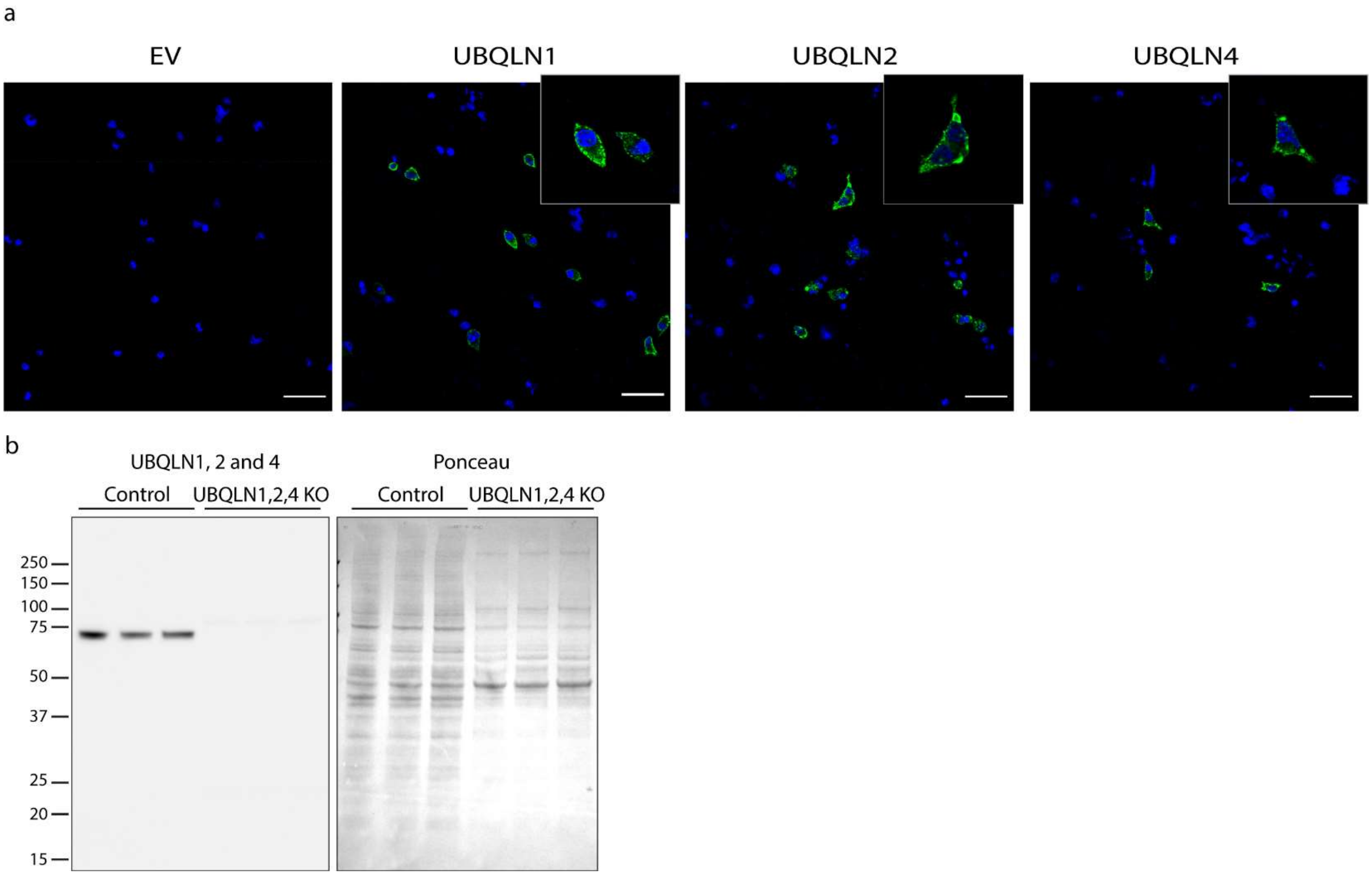

Supplementary Figure S2.

Immunofluorescence of FLAG-UBQLNs 1, 2 and 4 in UBQLN1, 2 and 4 triple knockout HEK-293 cells shows that all three ubiquilins are capable of forming puncta independent of endogenous expression of the other UBQLN proteins. Similar to results in control HEK-293 cells from Figure 3. Western blot of a control cell line compared to UBQLN1, 2 and 4 triple knockout cells using UBQLN1, 2 and 4-specific antibodies (left) and Ponceau (right).

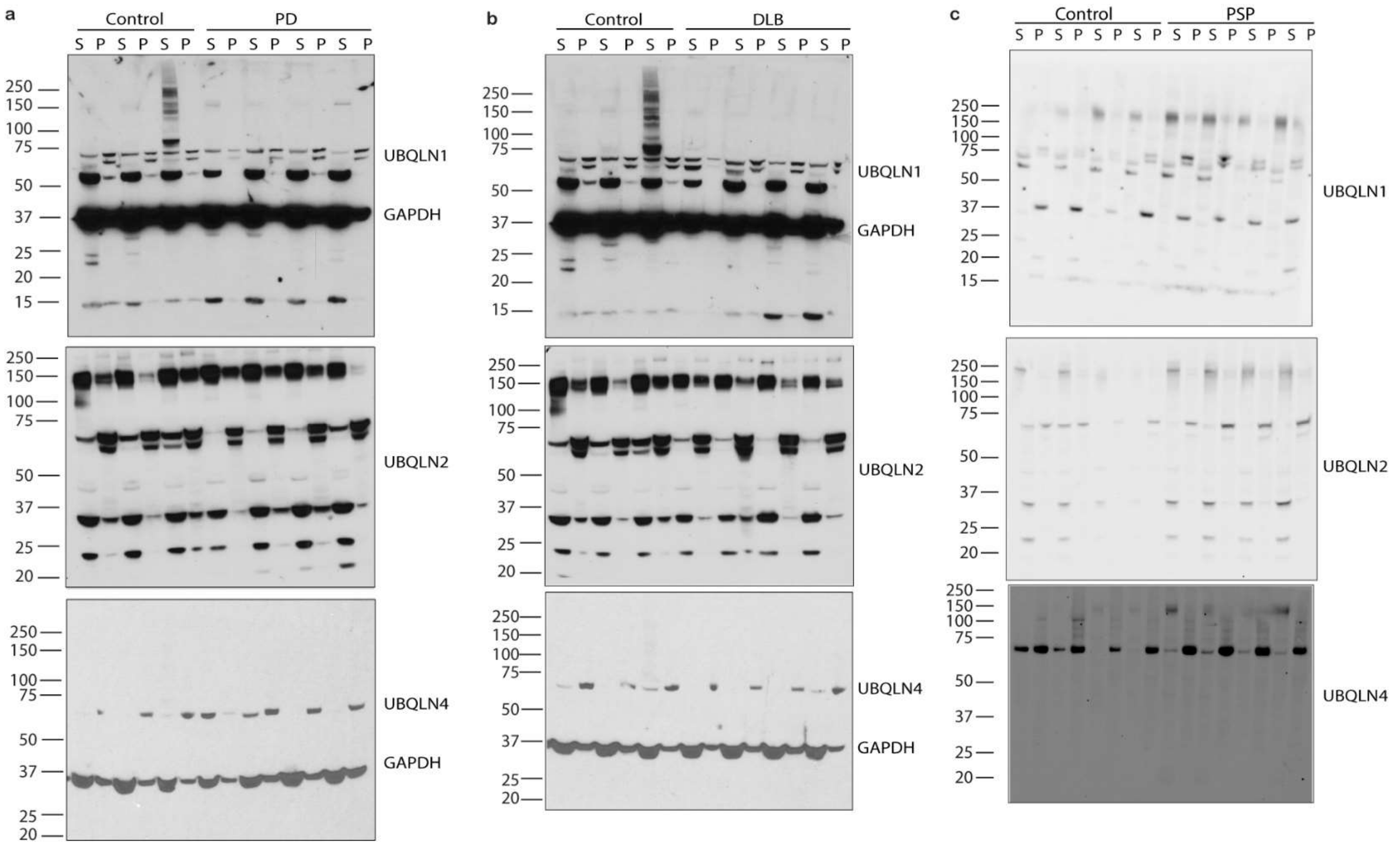

Supplemental Figure S3

Full, uncropped Western blots of human brain lysates from Figure 4 detected with UBQLN1, 2 and 4-specific antibodies.

Human Disease Tissue			
Diagnosis	Sex	Age	PMI* (hrs)
Control	Female	83	21
Control	Female	80	19
Control	Male	100	3
Control	Female	96	18
Control	Male	75	9
Control	Male	65	24
Control	Female	83	Unknown
Control	Male	71	4
Control	Female	80	5
Control	Male	65	14
Control	Female	74	6
Control	Female	76	14
Control	Male	83	28
PD	Male	78	22
PD	Male	74	14
PD	Female	71	7
PD	Male	86	10

PD	Female	74	6
PDD	Male	81	16
DLB	Male	78	12
DLB	Female	82	10
DLB	Male	84	5
DLB	Female	68	24
DLB	Male	66	8
DLB	Male	86	Unknown
DLB	Male	66	15
DLB	Male	71	5
DLB	Female	80	4
DLB	Female	57	9
DLB	Female	84	6
DLB	Male	87	13
DLB	Male	72	18
DLB	Female	71	12
PSP	Female	88	15
PSP	Male	64	4
PSP	Female	66	12
PSP	Male	66	5
PSP	Male	73	4
PSP	Male	77	12
PSP	Female	78	6
PSP	Male	73	4
PSP	Female	79	6
PSP	Male	79	6
PSP	Male	73	3
PSP	Female	54	12

Supplementary Table S1 Human samples used for analysis of UBQLNs. *PMI - postmortem interval.

ANOVA model F statistic and p-value: $F_{3,6} = 5.30, p = 0.025$

Y=UBQLN1 punctum area	Coefficients	Standard Error	t Stat	P-value
Intercept	31.8784	10.22984	3.116216	0.00291025
Circularity	-27.0624	11.75924	-2.30137	0.025184191

ANOVA model F statistic and p-value: $F_{3,6} = 8.72, p = 0.004$

Y=UBQLN2 punctum area	Coefficients	t Stat	P-value
Intercept	18.0250212	4.597055818	1.39259E-05
Circularity	-13.59759491	-2.9536822	0.004005986

ANOVA model F statistic and p-value: $F_{3,6} = 4.67, p = 0.034$

**Y=UBQLN4 punctum area**	**Coefficients**	**t Stat**	**P-value**
Intercept	22.76455303	3.689648536	0.000397739
Circularity	-15.95867673	-2.16149299	0.033503036

Supplementary Table S2. Linear regression analysis of punctum area using circularity as a predictor in the images taken from live HEK293-T cells transfected with the indicated eGFP-labeled ubiquilins.

Supplementary Movie 1: Fusion of eGFP-UBQLN1 puncta (scale bar 2.5µm)

Supplementary Movie 2: Fusion of eGFP-UBQLN1 puncta (scale bar 2.5µm)

Supplementary Movie 3: Fusion of eGFP-UBQLN2 puncta (scale bar 2.5µm)

Supplementary Movie 4: Fusion of eGFP-UBQLN2 puncta (scale bar 2.5µm)

Supplementary Movie 5: Fusion of eGFP-UBQLN2 puncta (scale bar 2.5µm)

Supplementary Movie 6: Fusion of eGFP-UBQLN4 puncta (scale bar 2.5µm)

Supplementary Movie 7: Fusion of eGFP-UBQLN4 puncta (scale bar 2.5µm)

Supplementary Movie 8: Fission of UBQLN4 puncta.
